# Phosphorylation of mRNA Decapping Protein Dcp1a by the ERK Signaling Pathway during Early Differentiation of 3T3-L1 Preadipocytes

**DOI:** 10.1371/journal.pone.0061697

**Published:** 2013-04-18

**Authors:** Pei-Yu Chiang, Yu-Fang Shen, Yu-Lun Su, Ching-Han Kao, Nien-Yi Lin, Pang-Hung Hsu, Ming-Daw Tsai, Shun-Chang Wang, Geen-Dong Chang, Sheng-Chung Lee, Ching-Jin Chang

**Affiliations:** 1 Graduate Institute of Biochemical Sciences, College of Life Science, National Taiwan University, Taipei, Taiwan; 2 Institute of Biological Chemistry, Academia Sinica, Taipei, Taiwan; 3 Department of Life Science, Institute of Bioscience and Biotechnology, National Taiwan Ocean University, Keelung, Taiwan; 4 Genomic Research Center, Academia Sinica, Taipei, Taiwan; 5 Institute of Molecular Medicine, College of Medicine, National Taiwan University, Taipei, Taiwan; German Cancer Research Center, Germany

## Abstract

**Background:**

Turnover of mRNA is a critical step in the regulation of gene expression, and an important step in mRNA decay is removal of the 5′ cap. We previously demonstrated that the expression of some immediate early gene mRNAs is controlled by RNA stability during early differentiation of 3T3-L1 preadipocytes.

**Methodology/Principal Findings:**

Here we show that the mouse decapping protein Dcp1a is phosphorylated via the ERK signaling pathway during early differentiation of preadipocytes. Mass spectrometry analysis and site-directed mutagenesis combined with a kinase assay identified ERK pathway–mediated dual phosphorylation at Ser 315 and Ser 319 of Dcp1a. To understand the functional effects of Dcp1a phosphorylation, we examined protein-protein interactions between Dcp1a and other decapping components with co-immunoprecipitation. Dcp1a interacted with Ddx6 and Edc3 through its proline-rich C-terminal extension, whereas the conserved EVH1 (enabled vasodilator-stimulated protein homology 1) domain in the N terminus of Dcp1a showed a stronger interaction with Dcp2. Once ERK signaling was activated, the interaction between Dcp1a and Ddx6, Edc3, or Edc4 was not affected by Dcp1a phosphorylation. Phosphorylated Dcp1a did, however, enhanced interaction with Dcp2. Protein complexes immunoprecipitated with the recombinant phosphomimetic Dcp1a(S315D/S319D) mutant contained more Dcp2 than did those immunoprecipitated with the nonphosphorylated Dcp1a(S315A/S319A) mutant. In addition, Dcp1a associated with AU-rich element (ARE)-containing mRNAs such as MAPK phosphatase-1 (MKP-1), whose mRNA stability was analyzed under the overexpression of Dcp1a constructs in the Dcp1a knockdown 3T3-L1 cells.

**Conclusions/Significance:**

Our findings suggest that ERK-phosphorylated Dcp1a enhances its interaction with the decapping enzyme Dcp2 during early differentiation of 3T3-L1 cells.

## Introduction

Turnover of mRNA is critical for regulating gene expression. In eukaryotes, mRNA decay is initiated by deadenylation and the activation of two major decay pathways from the 3′ to the 5′ end or from the 5′ to the 3′ end [1]. mRNAs are degraded from the 3′ end by the exosome, which is a large complex comprised of 10 or more 3′-to-5′ exonucleases. Alternatively, the 5′ cap is hydrolyzed by the decapping complex (Dcp1/Dcp2) to release m^7^GDP and 5′-monophosphorylated mRNAs, which are preferentially degraded by the 5′-to-3′ exonuclease Xrn1 [2], [3]. In addition to catalyzing mRNA turnover, decapping is a crucial step in several specialized eukaryotic mRNA decay pathways, such as nonsense-mediated decay, AU-rich element (ARE)-mediated decay, and the miRNA-mediated turnover of certain mRNAs [4]–[8].

The decapping process has been extensively studied in yeast, and several factors are involved, including the decapping enzyme Dcp2p, the decapping cofactor Dcp1p, the decapping activator Dhh1p (RCK/p54 or Ddx6 in mammals), Pat1p, Edc3p, the Lsm1p–7p complex, and Xrn1p [5], [9], [10]. These factors form macroscopic aggregates that appear as punctate cytoplasmic foci (100–300 nm) termed mRNA processing bodies (P-bodies) [9]–[12]. The sequences of the mammalian homologs Dcp1a and Dcp1b are similar to yeast Dcp1p in their N-terminal regions [13]. The N terminus of mammalian Dcp1a contains an enabled vasodilator-stimulated protein homology 1/Wiskott-Aldrich syndrome protein homology 1 (EVH1/WH1) functional domain, which is a protein-interacting domain that is conserved among eukaryotic Dcp1 proteins. Although mammalian Dcp1 homologs are conserved at the N terminus, the long C terminus of the mammalian Dcp1 homologs shows no similarity to yeast Dcp1p. Yeast Dcp1p can physically interact with Dcp2p, and recombinant Dcp1p can enhance the decapping activity of yeast Dcp2p [14], [15]. A similar interaction has not been observed in human cells, but Hedls/Ge-1/Edc4 is believed to be the essential factor responsible for the assembly of the decapping complex [16]–[18].

Recent kinetic and structural studies of yeast decapping complexes have provided clues to how Dcp2p activity is regulated by Dcp1p [19], [20]. Mammalian Dcp1a has little homology with yeast Dcp1p, and little is known about the details of the mammalian decapping complex assembly or the regulation of decapping activity. In this study, we observed that Dcp1a was phosphorylated in response to differentiation signals in 3T3-L1 cells. Using mass spectrometry (MS) analysis combined with *in vivo* and *in vitro* phosphorylation assays and site-directed mutagenesis, we identified Ser315 and Ser319 as being phosphorylated by the ERK signaling pathway. We also demonstrated that the physical interaction of Dcp1a with Dcp2 was enhanced by phosphorylation at Ser315 and Ser319. The possible functional effect of Dcp1a phosphorylation in regulating ARE-containing mRNA degradation was investigated in the early differentiation of 3T3-L1 cells.

## Results

### Dcp1a is phosphorylated via the ERK signaling pathway during early differentiation of 3T3-L1 preadipocytes

**Figure 1:**
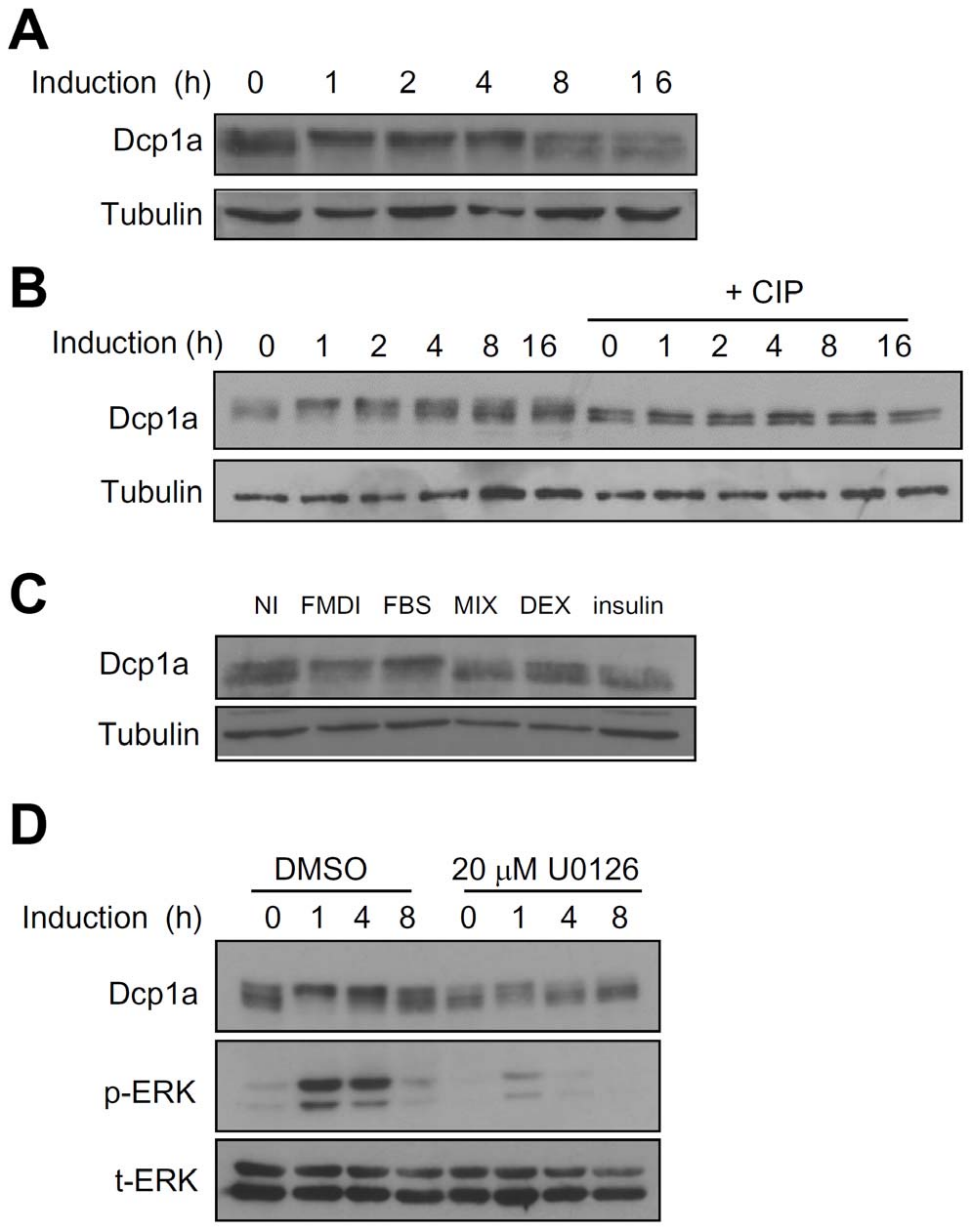
Dcp1a is phosphorylated during early differentiation of 3T3-L1 preadipocytes. (A) The expression profile of endogenous Dcp1a during the early differentiation of 3T3-L1 preadipocytes. Two days after reaching confluency, cultures of 3T3-L1 preadipocytes were induced to differentiate with an induction cocktail for 0, 1, 2, 4, 8, and 16 h. Cell extracts were isolated, and western blot analysis for detection of Dcp1a protein was conducted using 30 µg of each sample. Tubulin served as the protein input control. (B) CIP treatment. Confluent cultures of 3T3-L1 preadipocytes were induced to differentiate for 0–16 h, and cell extracts were isolated and treated with CIP. Western blot analysis was performed as in panel A. (C) Two days after reaching confluency, 3T3-L1 preadipocytes were induced to differentiate with individual components of the induction cocktail (FBS, MIX, DEX, insulin) or with the induction cocktail (FMDI) or non-induced control (NI). After incubating for 2 h, cell extracts were analyzed as in panel A. (D) Two days after reaching confluency, 3T3-L1 preadipocytes were pretreated with either DMSO (vehicle control) or 20 µM U0126 for 30 min before induction and then were induced with FMDI for 0, 1, 4, and 8 h in DMSO or U0126. Cell extracts were isolated for western blot analysis using anti-Dcp1a, anti-phospho-ERK (p-ERK), and anti-total ERK (t-ERK). All experiments were independently repeated three to five times with similar results, one of which is shown here.

We previously demonstrated that the mRNA expression of some immediate early genes (IEGs) such as tristetraprolin (TTP) and MAPK phosphatase-1 (MKP-1) is controlled by RNA stability during early differentiation of 3T3-L1 preadipocytes [21], [22]. We thus examined whether mRNA decapping machinery plays a role in the expression of these IEGs. 3T3-L1 preadipocytes were induced to differentiate by incubation with an adipogenic cocktail comprised of methylisobutylmethylxanthine (MIX), dexamethasone (DEX), bovine insulin, and fetal bovine serum (FBS) (FMDI). Interestingly, the expression profile analysis of decapping protein Dcp1a revealed that endogenous mouse Dcp1a consisted of multiple bands on western blots, with the lower bands being more prevalent when cells were not induced (Fig. 1A). As the preadipocytes started to differentiate, the upper bands of Dcp1a became predominant 1 to 4 h after induction. After 8 h of induction, the predominant forms of Dcp1a became the lower bands again. We then examined whether the shifted Dcp1a bands included phosphorylated Dcp1a by treating cell lysates from the differentiation time course with calf intestinal phosphatase (CIP) (Fig. 1B). CIP treatment resulted in a complete loss of the upper bands, suggesting that Dcp1a was phosphorylated and that the upper bands represented phosphorylated forms of Dcp1a. To identify the kinase responsible for Dcp1a phosphorylation, each inducer of differentiation was examined separately (Fig. 1C). Two days after reaching confluency, cultures were treated with FBS, MIX, DEX, insulin, or the complete induction cocktail, FMDI. Cultures treated with FMDI or FBS alone were activated and showed both forms of Dcp1a, but treatment with MIX, DEX, or insulin alone produced primarily the hypophosphorylated form of Dcp1a. Thus, we considered the FBS-responsive MAPK pathway to be a possible signal transduction cascade involved in the regulation of Dcp1a phosphorylation. To study this issue, the ERK pathway inhibitor U0126 was used to determine whether Dcp1a phosphorylation was regulated by ERK. Treatment with U0126 decreased the activation of ERK and caused Dcp1a to remain hypophosphorylated at 1 and 4 h after induction (Fig. 1D). Thus Dcp1a, one of the decapping complex components, was phosphorylated in response to preadipocyte differentiation signals.

### Ser315 and Ser319 of Dcp1a are phosphorylated by ERK

**Figure 2:**
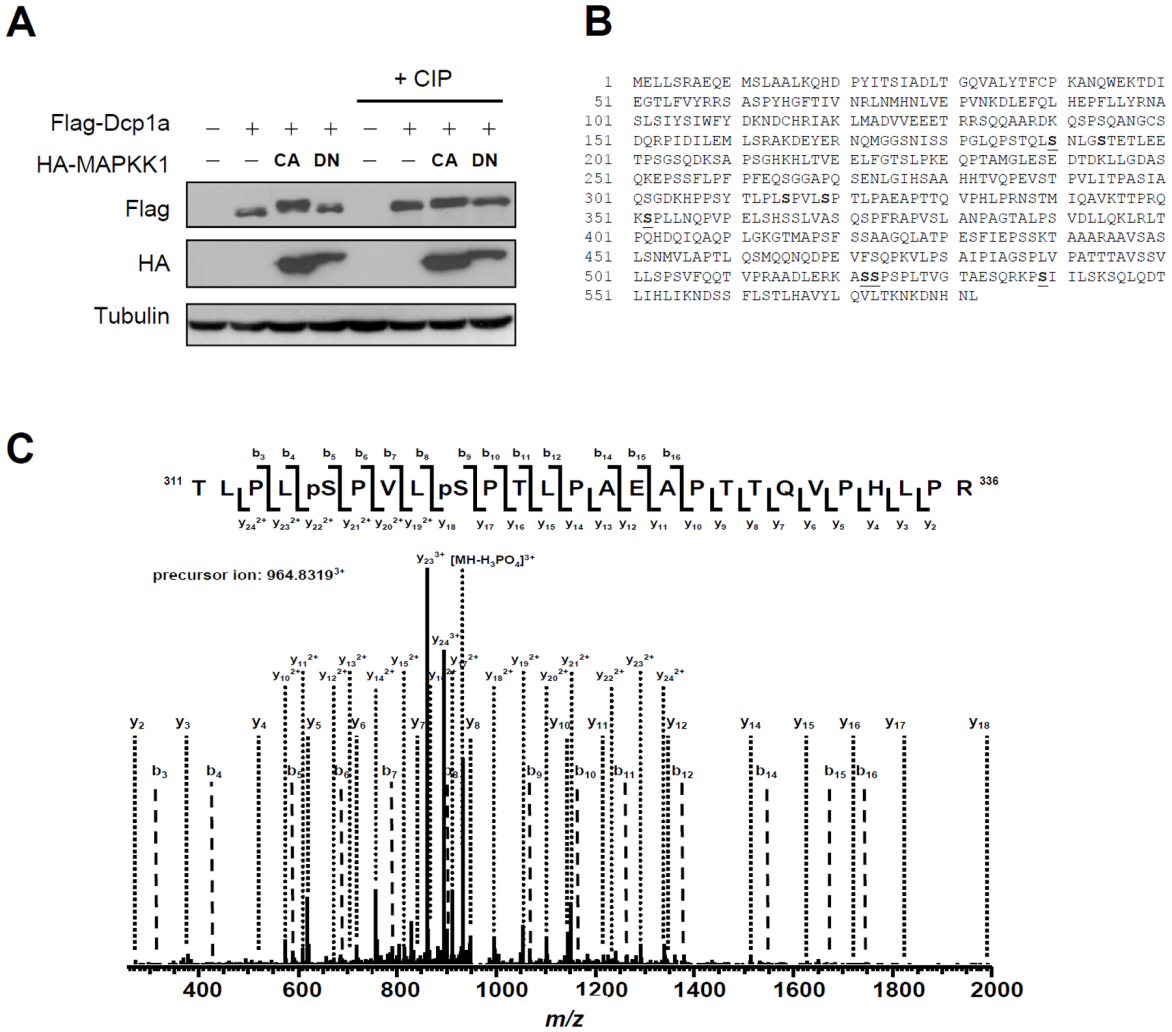
Two ERK phosphorylation sites at Ser315 and Ser319 of Dcp1a were identified with mass spectrometry (MS). (A) Flag-tagged Dcp1a was expressed in HEK 293T cells, either alone or together with CA or DN hemagglutinin (HA)-tagged MAPKK1 mutants. Protein samples after CIP treatment or from control conditions were analyzed with western blotting using anti-Flag, anti-HA, and anti-tubulin. (B) Phosphorylated residues of Dcp1a were identified using MS with DN MAPKK1 or CA MAPKK1. The amino acid sequence of Dcp1a and the identified phosphorylated residues from samples in the presence of CA MAPKK1 (red bold) or DN MAPKK1 (red underlined) are shown. Phosphorylated Ser315 and Ser319 were observed in Dcp1a only with CA MAPKK1 but not with DN MAPKK1. The other site, Ser194, is not a typical site for MAPK. (C) The MS/MS spectrum shows the concurrency of dual phosphorylation of Ser315 and Ser319 in Dcp1a.

**Figure 3:**
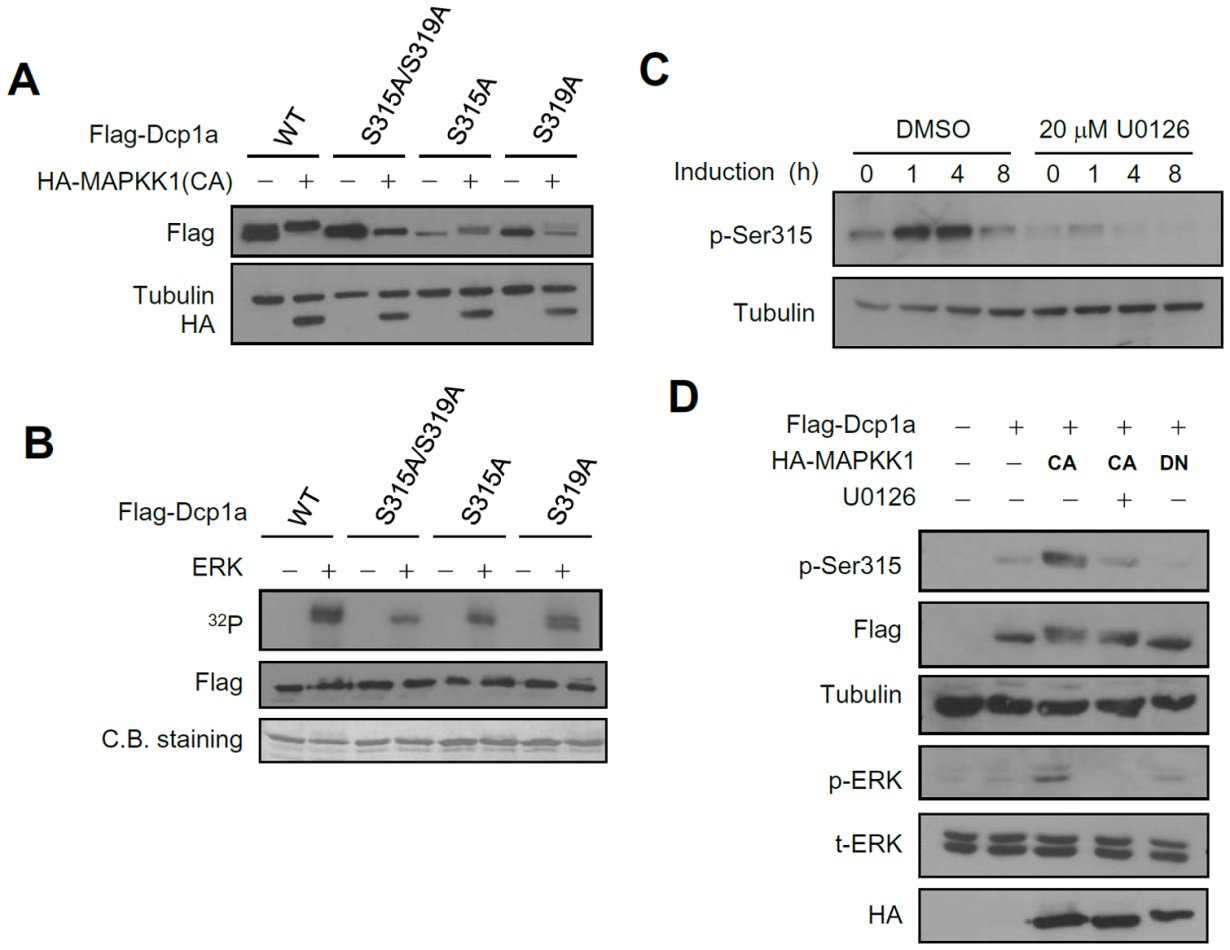
Phosphorylation analysis of Dcp1a mutants *in vivo* and *in vitro*. (A) The Dcp1a mutants S315A/S319A (double mutant), S315A, and S319A were co-transfected into HEK 293T cells with HA-tagged CA MAPKK1 for an *in vivo* phosphorylation assay. Protein samples were analyzed by western blotting using anti-Flag, anti-HA, and anti-tubulin. (B) *In vitro* phosphorylation assay of recombinant Dcp1a by ERK2. The recombinant wild-type and mutant Dcp1a proteins purified from *E. coli* were incubated with active ERK2 and [^32^P]ATP at 30°C for 30 min. The reaction mixtures were separated by SDS-PAGE and analyzed by autoradiography. The recombinant proteins used in the reaction were detected by western blotting with anti-Flag or Coomassie blue (C.B.) staining. (C) Western blotting analysis of 3T3-L1 proteins using anti-p-Ser315. The samples from Fig. 1D were analyzed with anti-p-Ser315 and anti-Tubulin. (D) p-Ser315 detection in over-expressed Dcp1a. Flag-tagged Dcp1a and HA-tagged MAPKK1 mutants (CA or DN) were co-expressed in HEK 293T cells. Cells were treated in the presence or absence of 10 µM U0126 for 30 min, and cell extracts were isolated for western blotting with anti-p-Ser315, anti-Flag, and anti-tubulin. One representative of three to five independent experiments with similar results is shown.

To identify the ERK-mediated phosphorylation residues of Dcp1a, Flag-Dcp1a was co-expressed with the constitutively active (CA) and dominant negative (DN) forms of the upstream kinase of ERK, MAPKK1, which were used for activation and inhibition of ERK, respectively. Western blotting analysis showed that CA MAPKK1 caused an upward mobility shift of Dcp1a on SDS-polyacrylamide gels, an effect that was absent following treatment with CIP, suggesting protein hyperphosphorylation (Fig. 2A). Flag-Dcp1a was immunoprecipitated (IP) with anti-Flag beads, eluted with flag peptides, and separated by SDS-PAGE. ERK-phosphorylated peptides were then analyzed with MS. The phosphorylated residues are shown in Fig. 2B. Phosphorylation of Ser315 and Ser319, which are typical phosphorylation sites for MAPK, was specific to the CA form of MAPKK1. The MS data also confirmed the presence of Ser315/Ser319 double phosphorylation (Fig. 2C). To confirm ERK phosphorylation of these two serine residues, we produced alanine mutants for *in vivo* and *in vitro* kinase assays. Phosphorylation of the individual S315A or S319A mutants did occur after co-transfection with the CA form of MAPKK1, as indicated by mobility shifting of double bands, whereas the S315A/S319A mutant showed only a single lower band (Fig. 3A). Next, recombinant Dcp1a mutants were purified from *E. coli* and incubated with active ERK2 for an *in vitro* phosphorylation assay. Consistent with the *in vivo* assay, two phosphorylation signals were observed in each single mutant, and the phosphorylation level of the S315A/S319A double mutant was decreased (Fig. 3B). A specific antibody against p-Ser315 was generated to detect phosphorylation of endogenous Dcp1a in preadipocytes undergoing early differentiation. Stronger signals were observed 1 and 4 h after induction than at 8 h after induction, and U0126 treatment dramatically decreased these signals (Fig. 3C). Consistent with this result, overexpression of Flag-Dcp1a produced a stronger signal from anti-p-Ser315 when co-transfected with the CA form of MAPKK1, whereas treatment with U0126 or co-transfection with the DN form of MAPKK1 decreased the signal (Fig. 3D). Together, our *in vitro* and *in vivo* kinase analyses and the results using the phospho-specific antibody demonstrated that Dcp1a was phosphorylated at Ser315 and Ser319 via ERK signaling.

Our mouse *Dcp1a* was cloned from the sequence predicted from the human homolog. Comparison of mouse *Dcp1a* and human *DCP1a* showed 90% similarity between their mRNA sequences (data not shown). We did, however, observe an upstream in-frame AUG start codon that allows the inclusion of an additional 20 amino acids in the N terminus of mouse Dcp1a [23] (Fig. S1A). To determine the exact start codon, we examined the expression of mouse Dcp1a using a specific antibody against this 20 amino acid–region and confirmed that like human Dcp1a, mouse Dcp1a is translated from the downstream AUG (Fig. S1B and S1C). Therefore, the construct for mouse Dcp1a expression that we used encodes a protein of 582 amino acids in length instead of 602 amino acids in length as predicted by the cDNA sequence in the database.

### C-terminal extension of Dcp1a is responsible for Ddx6 and Edc3 interaction, and the N-terminal EVH1/WH1 domain interacts with Dcp2

**Figure 4:**
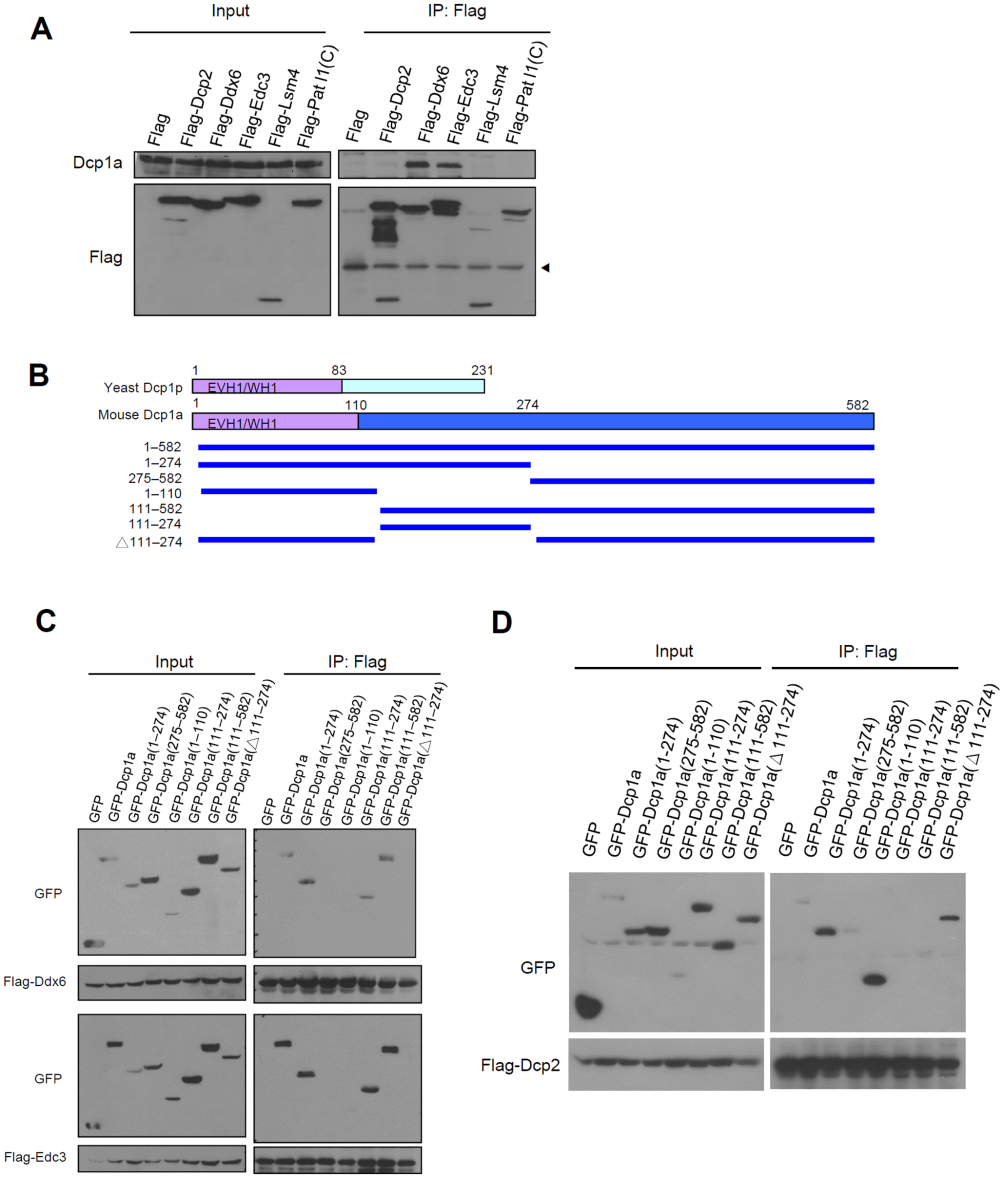
Mapping analysis of the interacting domain of Dcp1a with Ddx6, Edc3, and Dcp2. (**A**) Co-IP of endogenous Dcp1a with decapping complex components Ddx6 and Edc3. HEK293T cells were transiently transfected with the indicated Flag-tagged protein expression plasmids. IP was carried out using anti-Flag M2 agarose beads, and western blot analysis was conducted using anti-Dcp1a. The arrowhead indicates the immunoglobulin light chain. Patl1(C) indicates the C terminus of Patl1. One representative of three independent experiments with similar results is shown. (B) Schematic representation of yeast Dcp1p, mouse Dcp1a, and Dcp1a deletion constructs. (C) Amino acid region 111–274 of Dcp1a was required for association with Ddx6 and Edc3. GFP-Dcp1a deletion constructs, as indicated, were individually transfected with Flag-Ddx6 or Flag-Edc3 expression plasmids, and IP was carried out using anti-Flag M2 agarose beads. (D) The amino acids 1–110 of Dcp1a interact with Dcp2. Full-length GFP-Dcp1a and GFP-Dcp1a deletion constructs, as indicated, were co-expressed with Flag-Dcp2 in HEK 293T cells. Cell extracts were isolated, and IP was performed as described in (C). The left panels in (C) and (D) represent 10% of the input, and the right panels represent immunoprecipitated complexes. One representative of three independent experiments with similar results is shown.

Because Dcp1a is a cofactor for the decapping complex and can associate with other decapping components, we hypothesized that phosphorylation of Dcp1a may affect protein-protein interactions. To address this issue, co-IP was performed to study the physical interactions between Dcp1a and other decapping complex components, including Dcp2, Edc3, Ddx6, Lsm4, and the C terminus of Patl1. Ectopically expressed Ddx6 and Edc3 immunoprecipitated endogenous Dcp1a (Fig. 4A). In contrast, Dcp1a was not detected after co-IP with Dcp2, Lsm4, or the C terminus of Patl1. Compared with the yeast Dcp1p sequence, mouse Dcp1a contains a conserved N-terminal EVH1/WH1 domain and a variable proline-rich C-terminal extension (Fig. 4B). To define which regions of Dcp1a mediated the observed protein interactions, we constructed a series of Dcp1a deletion mutants, with deletions of amino acids 1–274 or 275–582, the highly conserved EVH1/WH1 domain (1–110), the ΔEVH1/WH1 domain (111–582), amino acids 111–274, and amino acids Δ111–274 (Fig. 4B). GFP-tagged Dcp1a deletion mutants and Flag-tagged Ddx6, Edc3, or Dcp2 were co-expressed in HEK 293T cells. Cell lysates were immunoprecipitated using M2 beads, and GFP served as a negative control. Input (5% of the whole-cell lysates) and immunoprecipitated proteins were analyzed by western blotting using anti-GFP and anti-Flag. GFP-Dcp1a(111–274) was co-immunoprecipitated with either Flag-Ddx6 or Flag-Edc3 (Fig. 4C). This result was confirmed by co-IP with the Δ111–274 mutant, which showed no interaction with Ddx6 or Edc3 (Fig. 4C). Although endogenous Dcp1a did not co-IP with Dcp2, the overexpressed full-length and C-terminal extension–deleted Dcp1a mutant, Dcp1a(1–110), did interact with Dcp2 (Fig. 4D). This region of Dcp1a contains an EVH1/WH1 domain that is conserved in yeast Dcp1p. Thus the proline-rich C-terminal extension of Dcp1a mediated the interaction with Ddx6 and Edc3 but did not interact with Dcp2. Our results also implied that the C-terminal extension of Dcp1a may play an important role in regulating Dcp1a-mediated interactions with other members of the decapping machinery.

### Phosphorylation-independent association between Dcp1a and Ddx6, Edc3, and Edc4

**Figure 5:**
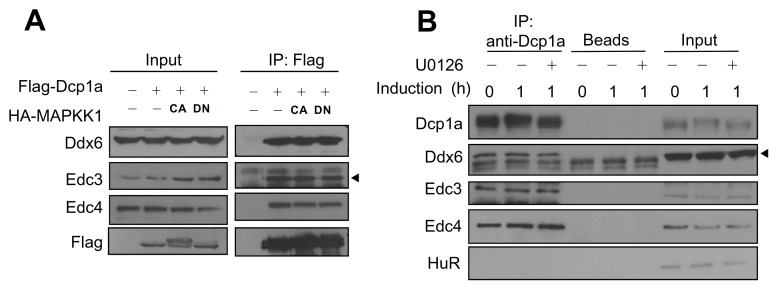
A phosphorylation-independent interaction between Dcp1a and decapping components and RNA. (A) Flag-tagged Dcp1a and HA-tagged CA or DN MAPKK1 mutants were co-expressed in HEK 293T cells. Cell extracts were immunoprecipitated using M2 beads. Input (5% of cell extracts; left panel) and immunoprecipitated protein complexes (right panel) were analyzed by western blotting using anti-Flag, anti-Ddx6, anti-Edc3, or anti-Edc4. The arrowhead indicates the specific Edc3 band. (B) Co-IP of Dcp1a and other decapping factors in 3T3-L1 cell extracts. Cell extracts from control cells or cells induced to differentiate for 1 h in the presence or absence of 20 µM U0126 were incubated with anti-Dcp1a and protein A–Sepharose. After extensive washing, the precipitated protein complexes were analyzed with western blotting with anti-Dcp1a, anti-Ddx6, anti-Edc3, anti-Edc4, and anti-HuR. The arrowhead indicates the specific Ddx6 band. Similar results were independently reproduced three times.

We further examined whether ERK-mediated Dcp1a phosphorylation modulates interactions between Dcp1a and other decapping components. Flag-tagged Dcp1a was ectopically expressed together with HA-tagged CA or DN MAPKK1 mutants in HEK 293T cells. After IP using M2 beads, protein complexes were detected with western blotting using anti-Ddx6, anti-Edc3, or anti-Edc4. Interactions between Dcp1a and Ddx6, Edc3, and Edc4 were not affected by the phosphorylation status of Dcp1a (Fig. 5A). Endogenous protein interactions were demonstrated by co-IP with extracts from control or 1-h differentiation-induced 3T3-L1 cells in the presence or absence of U0126. Protein complexes immunoprecipitated by anti-Dcp1a contained Ddx6, Edc3, and Edc4, but not HuR (negative control) (Fig. 5B). No significant differences were observed in the protein levels immunoprecipitated by Dcp1a with different phosphorylation states. Together, these results suggested that the phosphorylation of Dcp1a did not affect its association with Edc3, Edc4, or Ddx6. Moreover, immunofluorescence staining showed that phosphorylation of Dcp1a did not alter its subcellular localization or P-body formation in HeLa cells and 3T3-L1 cells (Fig. S2A and S2B).

### Phosphorylation of Dcp1a increases its association with Dcp2

**Figure 6:**
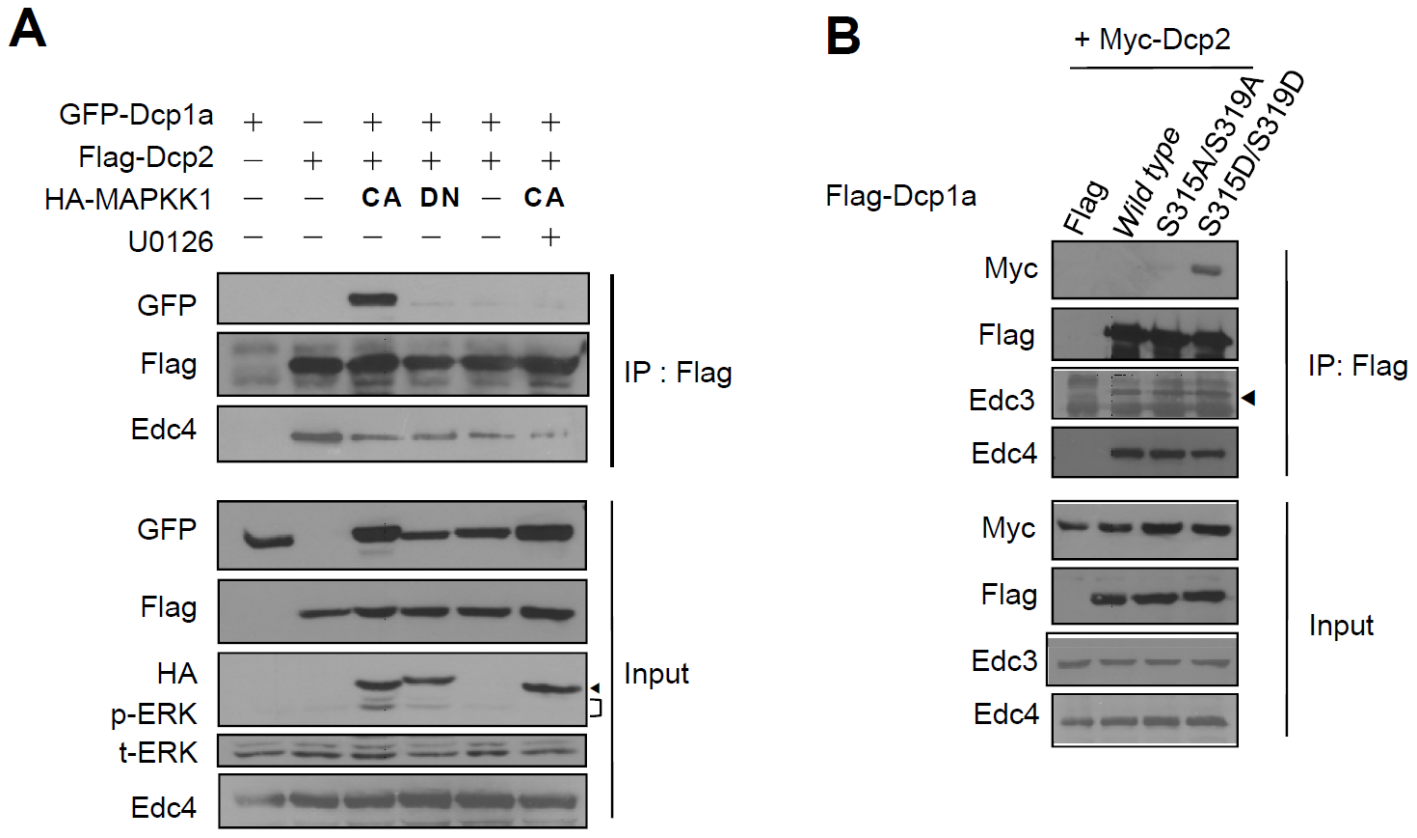
ERK-mediated phosphorylation of Dcp1a increases Dcp1a and Dcp2 association. (A) GFP-tagged Dcp1a, HA-tagged CA or DN MAPKK1 mutants, and Flag-tagged Dcp2 were co-expressed in HEK 293T cells. Protein complexes were immunoprecipitated using M2 beads. Inputs (50 µg of each cell extract) and immunoprecipitated protein complexes were analyzed using anti-Flag, anti-GFP, anti-HA, anti-ERK (t-ERK), anti-p-ERK, and anti-Edc4. The arrowhead indicates the HA MAPKK1 band, and the bracket indicates p-ERK signals. (B) Myc-tagged Dcp2 and Flag-tagged wild-type and mutant Dcp1a were co-expressed in HEK 293T cells. Inputs (50 µg of each cell extract) and immunoprecipitated protein complexes were used for western blotting with anti-Myc, anti-Flag, anti-Edc3, and anti-Edc4. The arrowhead indicates the specific Edc3 band. Similar results were independently reproduced five times.

Furthermore, we overexpressed Dcp1a and Dcp2 with different tags to perform co-IP experiments. Flag-tagged Dcp2 immunoprecipitated a greater amount of CA MAPKK1 induced phosphorylation of GFP-tagged Dcp1a in HEK 293T cells than did DN MAPKK1. This increased level was downregulated following treatment with U0126, whereas a similar amount of Edc4 was co-immunoprecipitated by Dcp2 in all reactions (Fig. 6A). This result indicated that the weak interaction between full-length Dcp1a and Dcp2 was strengthened by ERK-mediated phosphorylation of Dcp1a. In addition, we asked whether the S315D/S319D phosphomimetic Dcp1a mutant showed similar properties. We overexpressed Myc-tagged Dcp2 and Flag-tagged Dcp1a phosphorylation mutants in HEK 293T cells. The co-IP experiments demonstrated that the S315D/S319D double mutant showed an increased interaction with Dcp2 as compared with that observed with the S315A/S319A nonphosphomimetic Dcp1a mutant (Fig. 6B). The Edc3- and Edc4-interacting activity of Dcp1a was not affected by the mutations. The S315A/S319A mutant lost the response to CA MAPKK1 (Fig. S3), indicating that phosphorylation of the S315 and S319 residues is important for ERK-mediated Dcp1a activity. However, the cellular distribution or P body formation of S315A/S319A and S315D/S319D mutants was similar in HeLa cells (Fig. S4). Together, these results suggested that phosphorylation of Dcp1a at S315 and S319 via the ERK signaling pathway increased the interaction withDcp2.

### Association between Dcp1a and ARE-containing mRNAs

**Figure 7:**
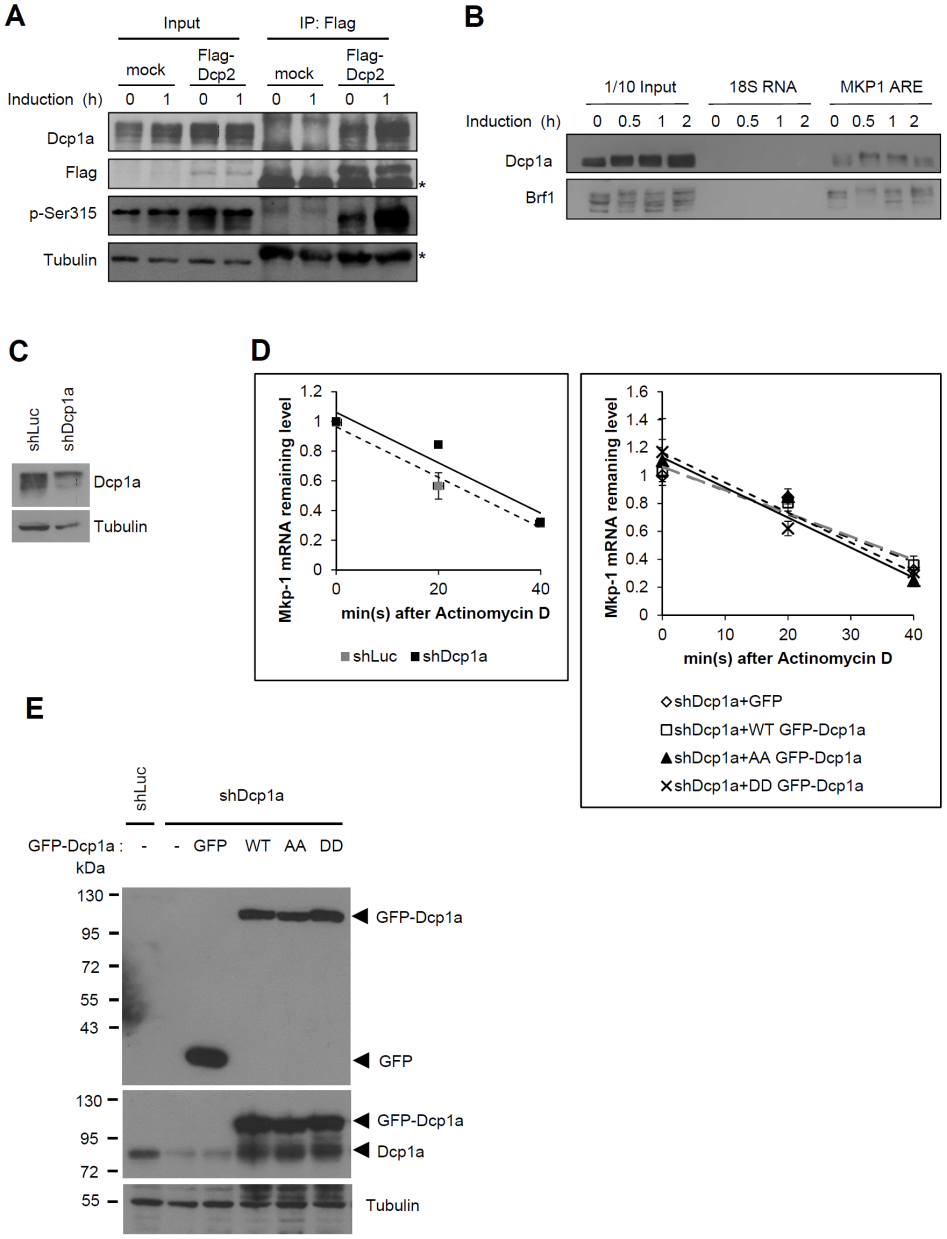
The physical and functional association between Dcp1a and ARE-containing mRNA. (A) Interaction between Dcp2 and Dcp1a in the extracts of 3T3-L1 cells. 70% confluent 3T3-L1 cells were transiently transfected with control vector or Flag-Dcp2 plasmids. After two days, the cells were untreated or treated with FMDI for 1 h and cell extracts were isolated for IP with anti-Flag M2 agarose beads. The precipitated protein complexes were western blotted with anti-Dcp1a, anti-Flag, anti-p-Ser315, and anti-tubulin. Input is 10% protein amount. (B) RNA pull-down. Cytoplasmic extracts from 3T3-L1 control cells or induced to differentiation for 0.5, 1 and 2 h were incubated with *in vitro* transcribed biotinylated MKP-1 ARE and control 18S RNA. The protein and biotinylated RNA complexes were recovered by addition of Streptavidin Sepharose. The brought-down complexes were resolved by gel electrophoresis followed by western blotting with anti-Dcp1a and anti-Brf1 antibodies. (C) Kockdown of Dcp1a in 3T3-L1 cells. The knockdown efficiency of Dcp1a was determined by western blotting with anti-Dcp1a and anti-tubulin. shLuc is a negative control. (D) Analysis of MKP-1 mRNA stability. The shLuc control cells, Dcp1a knockdown cells, and the Dcp1a knockdown cells overexpressed with wild-type (WT) or S315A/S319A (AA) or S315D/S319D (DD) GFP-tagged Dcp1a were induced by FMDI for 1 h, and then 10 µg/ml of actinomycin D was added for 0, 20 and 40 min. The RNA was isolated for quantitative PCR analysis by using primers of MKP-1 and actin. The graph displayed the MKP-1 mRNA remaining levels in the different treated cells as indicated. (E) The Dcp1a expression levels were detected by western blotting with anti-GFP (upper) and anti-Dcp1a (middle). The lower panel showed the tubulin expression levels as an internal control.

To demonstrate the interaction between Dcp1a and Dcp2 during early differentiation of 3T3-L1 cells, the cells were transiently transfected with Flag-Dcp2. After two days, the cells were induced with FMDI for 1 h and cell extracts were isolated for immunoprecipitation with anti-Flag (Fig. 7A). After 1 h induction, the Dcp2 would precipitate more endogenous Dcp1a. Moreover, Dcp1a is recruited by ARE-binding proteins including the TTP family proteins TTP and butyrate response factor 1 (BRF1), which are involved in the decay of ARE–containing mRNAs [4]. Thus, we examined whether Dcp1a can interact with ARE-containing mRNA such as MKP-1. RNA pull-down assay revealed that the biotin-labeled MKP-1 3′UTR could pull-down Dcp1a from 3T3-L1 cell extracts (Fig. 7B). To examine whether Dcp1a phosphorylation affects MKP-1 mRNA stability in 3T3-L1 cells, Dcp1a was knocked down by using lentivirus-carrying shRNA (Fig. 7C). The Dcp1a(WT), Dcp1a(S315A/S319A), and Dcp1a(S315D/S319D) were overexpressed, respectively, in the Dcp1a knockdown cells. The cells were treated with FMDI for 1 h to induce MKP-1 mRNA expression and actinomycin D was added to block transcription for 20 and 40 min. As shown in Fig. 7D left panel, knockdown of Dcp1a did not affect the MKP-1 mRNA stability. Moreover, there was no significant difference in MKP-1 mRNA half-life between ectopic expression of Dcp1a(S315A/S319A) and that of Dcp1a(S315D/S319D) (Fig. 7D right panel).The expression of endogenous Dcp1a and GFP-Dcp1a were detected by anti-Dcp1a and anti-GFP, respectively (Fig. 7E). Because the effective shRNA for Dcp1a knockdown is targeted to the coding sequence, the ectopic GFP-Dcp1a mRNA might compete with cellular Dcp1a mRNA and relieve the RNA interference to restore the endogenous Dcp1a expression (Fig. 7E middle panel).

## Discussion

In this study, two serines in Dcp1a, Ser315 and Ser319, were identified as ERK-mediated phosphorylation sites, and the presence of dual phosphorylated Dcp1a was critical for recruitment of the decapping enzyme Dcp2. First, we found that Dcp1a was hyperphosphorylated during early differentiation of 3T3-L1 preadipocytes (Fig. 1). The MAPK pathway was identified as a signal transduction cascade that participated in regulating Dcp1a phosphorylation. This finding was supported by showing that co-transfection with CA MAPKK1 resulted in alterations in the phosphorylation status of Dcp1a (Fig. 2). Second, Ser 315 and Ser 319 of Dcp1a were identified as the targets of ERK-mediated phosphorylation, which was confirmed by site-directed mutagenesis combined with kinase assays (Figs. 2 and 3). Using a phospho-specific antibody, we observed that phosphorylation at Ser315 was indeed enhanced following differentiation (Fig. 3C). Third, Dcp1a formed complexes with other decapping regulators, such as Ddx6, Edc3, and Edc4, via its proline-rich C-terminal domain (Fig. 4). Although Ser 315 and Ser 319 are located in the proline-rich C terminus of Dcp1a, interactions between Dcp1a and these decapping regulators were not altered by Dcp1a phosphorylation (Fig. 5). Consistent with this observation, confocal laser microscopy showed that Dcp1a was localized primarily in cytoplasmic processing bodies, and the phosphorylation status of Dcp1a did not affect its localization (Fig. S2). Phosphorylation of Dcp1a and the Dcp1a(S315D/S319D) phosphorylation mimic did, however, lead to increasing interactions with Dcp2 (Fig. 6). Thus, Ser315 and Ser319 in Dcp1a might be the essential phosphorylation sites for modulating decapping efficiency. Our results indicate that full-length Dcp1a might regulate the Dcp2-mediated mRNA decay when Dcp1a was phosphorylated via the ERK signaling pathway, but the Dcp1a-regulated mRNAs need to be further identified (Fig. 7).

In *Saccharomyces cerevisiae*, Dcp2p interacts directly with Dcp1p, and this interaction is required for decapping *in vivo* and *in vitro* [19], [20], [24]. The crystal structure of free Dcp2p from *Schizosaccharomyces pombe* indicates that the conserved Nudix domain forms a bi-lobed structure with an N-terminal α-helical domain, and this structure interacts with Dcp1p [25]. The Dcp1p-Dcp2p co-crystal structure exists in two conformations, open and closed, with the closed complex showing more catalytic activity [19]. A conformational change between the open and closed complexes may control decapping activity. Dcp1p increases the decapping activity of Dcp2p by enhancing and stabilizing the closed form, and this is responsible for the higher catalytic activity [26]. The interaction between mammalian Dcp1a and Dcp2 was observed in co-IP assay with overexpressed tagged proteins [13], [27]. However, Fenger-Gron *et al* displayed that human DCP1a does no IP human DCP2, and their association was only detected in the presence of EDC4 protein [16]. There were no results showing the interaction between the endogenous Dcp1a and Dcp2 in these previous reports. Our co-IP assay revealed that Flag-Dcp2 can not IP endogenous Dcp1a in 293T cells (Fig. 4A), whereas Flag-Dcp2 can associate with Dcp1a in 3T3-L1 cells (Fig. 7A). It might be due to the differential phosphorylation of serine 315 in these two cells (Fig. 3C and 3D). When Dcp1a was ectopically expressed, we observed that Flag-Dcp2 can IP GFP-Dcp1a (Fig. 4D). In Fig. 6A, the interaction of Dcp2 and Dcp1a was dependent on Dcp1a phosphorylation. The differential protein interactions of yeast Dcp1p and mammalian Dcp1a may be explained by their sequence differences. The sequences of human and mouse Dcp1a are highly homologous and contain a conserved EVH1/WH1 domain (amino acids 1–110) that shares 26% identity with the N terminus of yeast Dcp1p. In addition, Dcp1a has a long additional proline-rich sequence in the C terminus (amino acids 111–582), which was recently identified as containing two special domains: an MI (motif I) domain for protein interaction (amino acids 155–168) and a TD (trimrtization domain) for self-trimerization (amino acids 539–582) [27]. Previous reports showed that C-terminal deletion mutants of human DCP1a have impaired decapping activity [13] and that the amino acid motif 155–168 is required for interactions between Edc3 and Ddx6 [27]. Consistent with these observations, our data showed that a peptide containing amino acids 111–274 of Dcp1a was essential for interactions with Ddx6 and Edc3. The C terminus of Dcp1a also appeared to interfere with interactions with the decapping enzyme Dcp2 (Fig. 4D). Full-length Dcp1a interacted weakly with Dcp2, but a construct containing the EVH1/WH1 domain alone restored interactions with Dcp2, similar to interactions observed with Dcp1p. As EVH1/WH1 may function as a proline-rich binding domain [28], [29], we suggest the N-terminal EVH1/WH1 domain may be blocked by interactions with the C-terminal proline-rich extension. These observations imply that the recently evolved C-terminal extension of Dcp1a may play an essential role not only in interacting with decapping machinery components but also in regulating the functional communication between the N terminus and C terminus of Dcp1a.

Higher eukaryotes may require additional factors for the interaction between Dcp1a and Dcp2. Hedls/Ge-1/EDC4/VARICOSE was identified as being responsible for this interaction in human and plant [16]–[18]. In a recent report, amino acids 539–582 of human Dcp1a were shown to comprise a trimerization domain, which is required for interactions with human Edc4 as well as Dcp2 [27]. Edc4 seems to serve as a platform to link these two decapping proteins [16]. In our study, Dcp1a phosphorylation was able to compensate for the Edc4 effect to enhance the interaction with Dcp2. We suggest, under normal cellular conditions, Dcp1a is associated with Edc3, Ddx6, and Edc4, and the C-terminal proline-rich domain may interact with the N-terminal EVH1 domain. Once the ERK signaling pathway is activated by extracellular signals, such as an adipocyte differentiation signal, Ser315 and Ser319 of Dcp1a become phosphorylated, enabling Dcp1a to recruit Dcp2. Our MS data suggest that the two sites can be detected at the same time *in vivo*, and the importance of multiple phosphorylated residues at a phosphorylation site cluster has recently received increasing attention [30], [31]. A relationship between trimerization and phosphorylation of mouse Dcp1a is possible but requires verification. How phosphorylation of Dcp1a regulates the recruitment of Dcp2 is still unknown, but we hypothesize that the Dcp1a C terminus evolved in higher eukaryotes to respond to environmental changes to regulate the assembly of the mRNA decapping complex. In addition, other components of the mRNA decapping complex, including yeast Dcp2p and human Edc3, are also phosphorylated in response to extracellular signals [32], [33]. Recently, DCP1a was identified to be phosphorylated at serine 315 by JNK during IL-1 stimulation to control formation of P bodies [34]. They revealed phosphorylated DCP1a would increase IL-8 mRNA stability but not IκBα mRNA. It indicates that Dcp1a may respond to different signals and be phosphorylated by different kinases to modulate specific mRNA stability, which might be through interaction with specific RNA-binding proteins. These results suggest that the function of P-bodies is rapidly regulated by protein phosphorylation.

The induction of Dcp1a phosphorylation has been found not only during the early differentiation of 3T3-L1 preadipocytes but also during brain development, neuronal differentiation, cell division, oocyte maturation, and in response to cellular stresses [34]–[37]. In the growth-arrested state, 3T3-L1 preadipocytes synchronously re-enter the cell cycle and initiate mitotic clonal expansion after differentiation is induced. This is followed by the expression of genes that confer the adipocyte phenotype [38], [39]. During early differentiation of 3T3-L1 preadipocytes, several IEGs are induced [39]. One of them, TTP, was observed to bind AREs and thereby induce the rapid degradation of IEG mRNAs [21], [22]. The other TTP family member, BRF1, is constitutively expressed in 3T3-L1 cells [40]. Although TTP and BRF1 interact with Dcp and Dcp-associated subunits during ARE-mediated mRNA decay [4], [16], and the degradation of IEG mRNAs coincides with the phosphorylation profile of Dcp1a during early differentiation [22], we did not observe the alteration of mRNA half-life in ARE-containing mRNA in Dcp1a knockdown 3T3-L1 cells. ERK activation is required for 3T3-L1 differentiation [41]–[43]. ERK signaling activates the translation machinery to enhance the mitotic clonal expansion process [44]. At the same time, ERK also may activate the decapping machinery by phosphorylating Dcp1a to modulate gene expression. In the recent report, inhibiting accumulation of DCP1A and DCP2 in oocytes decreases not only degradation of mRNAs during meiotic maturation but also transcription of the zygotic genome [37]. Thus, the functional targets of Dcp1a and the effect of Dcp1a phosphorylation should be further investigated.

## Materials and Methods

### Ethics statement

We adhered to all institutional guidelines on the ethical use of animals in research. The protocols for the use of animals were approved by the Academia Sinica IACUC. All efforts were made to minimize pain and suffering.

### Plasmid constructs

Mouse cDNA was isolated from RAW264.7 macrophages, and full-length Dcp1a, Dcp2, Edc3, Ddx6, Lsm4, and the C terminus of Patl1 were PCR amplified and cloned into pCRII-TOPO plasmid (Invitrogen). After sequence confirmation, the fragments were sub-cloned into the mammalian cell expression vectors pCMV-Tag2B, pCMV-Tag3B (both from Stratagene), and pEGFP-C2 (Clontech). Dcp1a was also cloned into plasmid pQE81L-Flag (QIAGEN) for recombinant protein expression in *E. coli*. Dcp1a deletion constructs were cloned using the same methodology. PCR primers are listed in Table S1. Two HA-tagged MAPKK1 constructs, constitutively active (CA; R4F) and dominant negative (DN; K97M), were kindly provided by Dr. N. G. Ahn (University of Colorado, Boulder, Colorado, USA).

### Site-directed mutagenesis

Dcp1a mutants were generated using a QuikChange site-directed mutagenesis kit (Stratagene). Plasmids pCMV-Tag2-Dcp1a and PQE81L-Dcp1a-Flag were used as templates. Synthetic oligonucleotide primers were designed that were complementary to the opposite strand of the plasmids and that contained the desired mutation (Table S1). The reaction mixture contained 2.5 µl 10× reaction buffer, 10 ng template DNA, 62.5 ng each sense and antisense primer, 40 µM dNTPs, and 0.5 µl *Pfu ultra* DNA polymerase. The cycle conditions were one cycle of denaturation at 95°C for 5 min, 18 cycles of denaturation at 95°C for 1 min, annealing at 55°C for 1 min, elongation at 68°C for 7 min, and one cycle of elongation at 68°C for 5 min. *Dpn*I (1 µl; 10 U/µl) was added (target sequence: 5′-Gm6ATC-3′) to digest the parental DNA template at 37°C for 1 h. The reaction mixture (10 µl) was then transformed into competent bacteria (DH5α). All mutated plasmids were verified by sequence analysis.

### Cell culture

All cell lines were purchased from ATCC (Manassas, VA). HEK 293T and HeLa cells were cultured in DMEM (Gibco-BRL) supplemented with 3.7 g/l sodium bicarbonate and 10% Gibco qualified FBS (Gibco-BRL) in a 5% CO_2_ humidified incubator at 37°C. 3T3-L1 preadipocytes were cultured in DMEM supplemented with 1.5 g/l sodium bicarbonate and 10% bovine serum (Gibco-BRL) in a 5% CO_2_ humidified incubator at 37°C. Two days after reaching confluency, 3T3-L1 cells were stimulated to undergo differentiation into adipocytes by replacing fresh medium with an induction cocktail, FMDI, that was comprised of 10% FBS (HyClone-Characterized), 0.5 mM MIX (Sigma-Aldrich), 5 µM DEX (Sigma-Aldrich), and 1.7 µM bovine insulin. In the experiments with ERK kinase inhibitors, 3T3-L1 cells were pre-treated for 30 min with either 20 µM U0126 (Sigma-Aldrich) or the same volume of DMSO as a vehicle control 2 days after reaching confluency.

### Cell extract preparation

Harvested cells were lysed with NET buffer (50 mM Tris, pH 7.5; 150 mM NaCl; 1 mM EDTA; 0.1% Triton X-100) containing protease (Sigma-Aldrich) and phosphatase inhibitor (10 mM β-glycerol phosphate; 0.1 mM Na_2_MoO_4_; 0.1 mM Na_3_VO_4_, pH 10.0; 10 mM NaF) cocktails. For the CIP experiments, phosphatase inhibitor was not added to the NET buffer, and the cell lysates were treated with CIP at 37°C for 3 h.

### Co-IP assays

HEK 293T cells were co-transfected with the indicated plasmids using the CaPO_4_ precipitation method. Cells were harvested in NET buffer 24 h after transfection and were centrifuged at 15,000× *g* for 10 min. The supernatants were immunoprecipitated using anti-Flag M2 agarose (Sigma-Aldrich) at 4°C for 2 h. After the immunoprecipitation mixture was washed three times with NET buffer, bound proteins were eluted by boiling in protein sample buffer, and western blot analysis was carried out (see below). On the other hand, 3T3-L1 cells were induced to differentiate for 0 or 1 h, and cell lysates were harvested with NET buffer. Anti-Dcp1a was used to IP decapping protein complexes. For MS analysis, IP was conducted using samples from six dishes of HEK 293T cells that were transfected with pCMV-Flag-Dcp1a and HA-tagged MAPKK1 mutants. Bead-bound Flag-Dcp1a was eluted with Flag peptide (0.5 mg/ml; Sigma) and separated with SDS-PAGE. The proteins were stained with Coomassie blue, and bands were cut from the gel and digested with trypsin (Promega) for further MS analysis [45].

### SDS-PAGE and western blotting analysis

Western blot analysis was performed after electrophoretic separation of polypeptides with SDS-PAGE and transfer to Immobilon P transfer membrane (Millipore). Western blotting was conducted using the following specific antibodies: anti-Flag M2 (Sigma), anti-Myc (Genscript), anti-HA (Santa Cruz Biotechnology), anti-GFP (Santa Cruz Biotechnology), anti-Dcp1a (Abcam), anti-Dcp2 (Abcam), anti-Edc3 (Abcam), anti-Edc4 (Abcam), and anti-Ddx6 (Abcam). Membranes were incubated with the antibodies at room temperature for 1 h at the manufacturer's suggested dilution. The secondary antibody, horseradish peroxidase–conjugated goat anti–rabbit IgG (KPL) or horseradish peroxidase–conjugated goat anti–mouse IgG (KPL), was then added at room temperature for 1 h. Development was performed using ECL (PerkinElmer).

### Liquid chromatography (LC)-MS/MS analysis

Nano-LC–MS/MS experiments were performed on a LTQ-FT mass spectrometer (Thermo Fisher Scientific, Inc.) equipped with a nanoelectrospray ion source (New Objective, Inc.) in positive ion mode. Enzyme-digested cross-linked protein samples were injected onto a self-packed precolumn (150 µmI.D.×20 mm, 5 µm, 200 Å). Chromatographic separation was performed on a self-packed reversed phase C18 nanocolumn (75 µm I.D.×300 mm, 5 µm, 100 Å) using 0.1% formic acid in water (mobile phase A) and 0.1% formic acid in 80% acetonitrile (mobile phase B). A linear gradient from 5 to 40% mobile phase B for 40 min at a flow rate of 300 nL/min was applied. A scan cycle was initiated with a full-scan survey MS spectrum (mass range of 300–2000 Da) performed on the FT-ICR (Fourier Transform Ion Cyclotron Resonance) mass spectrometer with resolution of 100,000 at 400 Da. The ten most abundant ions detected in this scan were subjected to an MS/MS experiment performed in the linear ion trap. Ion accumulation (Auto Gain Control target number) and maximal ion accumulation time for full-scan and MS/MS were set at 1×10^6^ ions, 1000 ms and 5×10^4^ ions, 200 ms, respectively. Ions were fragmented with collision-induced dissociation with a normalized collision energy of 35%, activation Q of 0.3, and activation time of 30 ms. For data analysis, all MS/MS spectra were converted to mzXML and mgf format from the experimental RAW file with MM File Conversion Tools ([46], http://www.massmatrix.net), and then analyzed by MassMatrix [41] for MS/MS ion search. The search parameters in MassMatrix including the error tolerance of precursor ions and the MS/MS fragment ions in spectra were 10 ppm and 0.6 Da, and the enzyme was assigned to be trypsin with the missed cleavage number of three. The variable post-translational modifications in the search parameters were assigned to include the oxidation of methionine and the phosphorylation of serine/threonine/tyrosine.

### Expression and purification of recombinant proteins

BL21 (DE3) bacterial cells were transformed with PQE81L-Dcp1a-Flag or other Dcp1a mutants (S315A, S319A, S315A/S319A) and cultured at 30°C. After induction by incubating cells at 30°C with 0.1 mM IPTG for 4 h, the cells were harvested and lysed with lysis buffer (50 mM Tris-HCl, pH 7.0; 300 mM NaCl) and sonicated on ice for 20 min. Supernatants were collected after centrifugation, and Ni-NTA resin (QIAGEN) was added. The resin was poured into a solid support column and washed with 50 column volumes of wash buffer (50 mM Tris-HCl, pH 7.0; 500 mM NaCl; 10 mM imidazole) followed by a wash with two column volumes of PBS. Protein was eluted with PBS containing 200 mM imidazole. Anti-Flag M2 beads (Sigma-Aldrich) were used for the secondary purification. Protein was finally eluted with deionized water containing 0.5 mg/ml Flag peptide (Sigma-Aldrich).

### In vitro phosphorylation assay

A reaction cocktail containing 2 µg recombinant protein, 3 µl 10× reaction buffer, 1 µl [γ-^32^P]ATP (3000 µCi/mmol), and 20 U p42 MAP kinase (ERK2; New England Biolabs) in a final volume of 30 µl was incubated at 30°C for 30 min. The reactions were stopped by adding protein sample buffer. Samples were subjected to SDS-PAGE and observed by autoradiography.

### Generation of rabbit anti-p-Ser315 of Dcp1a

A peptide containing the sequence surrounding p-Ser315 of Dcp1a (YTLPLpSPVLSP) was coupled to keyhole limpet hemocyanin and used to immunize New Zealand white rabbits. Dcp1a/S315 phospho-specific antibodies were purified by peptide-affinity chromatography.

### RNA pull-down assay

Biotin-labeled RNAs containing *Mkp-1* ARE or 18S rRNA were transcribed *in vitro* using the T7-MEGAshortscript™ High Yield Transcription kit (Ambion). Cytoplasmic extracts of 3T3-L1 cells were prepared in binding buffer containing 10 mM HEPES, pH 7.5, 90 mM potassium acetate, 1.5 mM magnesium acetate, 2.5 mM DTT, 0.05% NP-40, and protease inhibitor cocktails. After the addition of 0.1 U/µl RNase inhibitor (Promega) and 20 µg/µl yeast tRNA (Ambion), lysates were absorbed by heparin-agarose (Sigma-Aldrich) and streptavidin-Sepharose (Invitrogen) beads for approximately 1 h at 4°C. After brief centrifugation, the supernatant was collected and total protein was quantified using the Bradford reagent. The same amount of total protein was incubated with biotin-labeled RNA probe and streptavidin-Sepharose for 3 h at 4°C. After washing five times with the binding buffer, the pulled-down RNA-protein complexes were subjected to immunoblotting with anti-Dcp1a and anti-Brf1.

### Lentivirus-mediated knockdown of Dcp1a

Lentivirus carrying pLKO.1-shRNA was produced in HEK 293T cells transfected with pCMVΔ8.91, pMD.G, and pLKO.1-shRNA. Mouse shDcp1a and the control shLuc which targeted *Luciferase* were purchased from the National RNAi Core Facility of Academia Sinica (Taipei, Taiwan) The target sequence of mouse shDcp1a is 5′- CCTCGGAATAGCACCATGATA-3′ (TRCN0000096665). Mouse 3T3-L1 cells were infected with the lentivirus for 48 h, and then infected colonies were selected with puromycin. The knockdown efficiency of shDcp1a was confirmed by probing with anti-Dcp1a antibody.

### mRNA stability analysis

The control and Dcp1a knockdown 3T3-L1 cells were overexpressed with CMV-driven wild-type or S315A/S319A or S315D/S319D GFP-tagged Dcp1a and induced endogenous MKP-1 mRNA expression by treatment with FMDI for 1 h. Then, 10 µg/ml actinomycin D was added to stop RNA synthesis for 0, 20, and 40 min. Total RNA was isolated with TRIzol reagent (Invitrogen) for reverse transcription. Real-time PCR was performed with the Applied Biosystems 7300 Real-Time PCR System (Applied Biosystems) in a total volume of 20 µl. Expression of MKP-1 and actin was analyzed in 3T3-L1 cells, and the expression of luciferase and actin was analyzed in HEK 293T cells using SYBR Green PCR Master Mix (Applied Biosystems) containing 50 ng cDNA and 160 nM each primer. The PCR amplification conditions were 40 cycles of 95°C for 15 s and 60°C for 1 min. The real-time PCR data were analyzed using the 2^−ΔΔCT^ relative quantification method, according to the manufacturer's directions. The average value ± SD was shown from three independent experiments.

### Statistical analysis

All of the data are presented as the mean ± SD of at least three independent experiments. The statistically significant values were determined by one-tailed Student's *t*-test.

## Supporting Information

Figure S1 Identification of a possible translation initiation codon in mouse DCP1a. (A) The nucleotide sequences of *Dcp1a* in mouse and human both contain two start codons that are 60 nucleotides apart. Human *DCP1a* has an in-frame stop-codon for the first AUG, but mouse *Dcp1a* does not. (B) Comparison of endogenous Dcp1a expression in mouse and human cells. 3T3-L1 (mouse) cell cultures were induced to differentiate for 0 and 2 h (lanes 1 and 2). HeLa (human) cells were serum starved overnight followed by induced for 2 h with Gibco Qualified FBS (lane 4), and untreated cells served as a control (lane 3). The remaining lanes (lanes 5–8), show cell extracts from lanes 1–4 were treated with 10 U CIP at 37°C for 1 h. (C) A rabbit polyclonal antiserum (N-20) specifically recognizing the first 20 amino acids of the predicted full-length mouse Dcp1a was generated. Antibody specificity (right panel) was shown by transient transfection of the vector control, Flag-tagged full-length Dcp1a (1–602), or Flag-tagged Dcp1a that begins at the second AUG (Dcp1a). Expression of endogenous Dcp1a (left figure) in 3T3-L1 cells was not detected by the antiserum. This implies that the translation of the Dcp1a mRNA started from the second AUG in the sequence.
(TIF)

Figure S2 Indirect immunofluorescence of Dcp1a in cells. (A) HeLa cells cultured in DMEM with 10% FBS on 3.5-cm dishes were transfected with GFP-Dcp1a (co-transfection with CA or DN MAPKK1). Cells were immunostained 1 day after transfection. Cells were rinsed briefly in PBS and fixed with 2% formaldehyde in PBS at room temperature for 20 min. HA-tagged MAPKK1 was stained with anti-HA followed by Alexa 594–conjugated anti-mouse, and the nuclei were stained with DAPI. The cells were visualized with confocal laser microscopy. (B) Two days after reaching confluency, cultures of 3T3-L1 preadipocytes were non-induced or induced with an induction cocktail FMDI for 1 h. Cells were fixed and immunostained with anti-Dcp1a followed by Alexa 594-conjugated anti-mouse and anti-p-Ser315 followed by Alexa 488-conjugated anti-rabbit, and the nuclei were stained with DAPI. The cells were visualized with confocal laser microscopy.
(TIF)

Figure S3 ERK signal can not further enhance the interaction between S315A/S319A Dcp1a mutant and Dcp2. HEK 293T cells were transfected with Flag-Dcp1a(wild type [WT]), HA-tagged CA MAPKK1, Flag-Dcp1a(S315A/S319A), Flag-Dcp1a(S315D/S319D), and Myc-Dcp2 as indicated. Protein complexes immunoprecipitated by anti-Flag wereanalysed by western blotting with indicated antibodies. The arrowhead indicates the non-specific band.
(TIF)

Figure S4 The similar cellular distribution of WT, S315A/S319A, and S315D/319D Dcp1a. HeLa cells were transfected with GFP-Dcp1a combined with Flag-Dcp1a mutant as indicated. Cells were immunostained 1 day after transfection with anti-Flag followed by Alexa 594–conjugated anti-mouse, and the nuclei were stained with DAPI. The cells were visualized with confocal laser microscopy.
(TIF)

Table S1 PCR and site-directed mutagenesis primer list.
(TIF)
