# Supplementary figures and images for: Phosphorylation of mRNA Decapping Protein Dcp1a by the ERK Signaling Pathway during Early Differentiation of 3T3-L1 Preadipocytes
Source: PLoS One. 2013 Apr 18;8(4):e61697. doi: 10.1371/journal.pone.0061697 (PMC3630112; doi:10.1371/journal.pone.0061697)

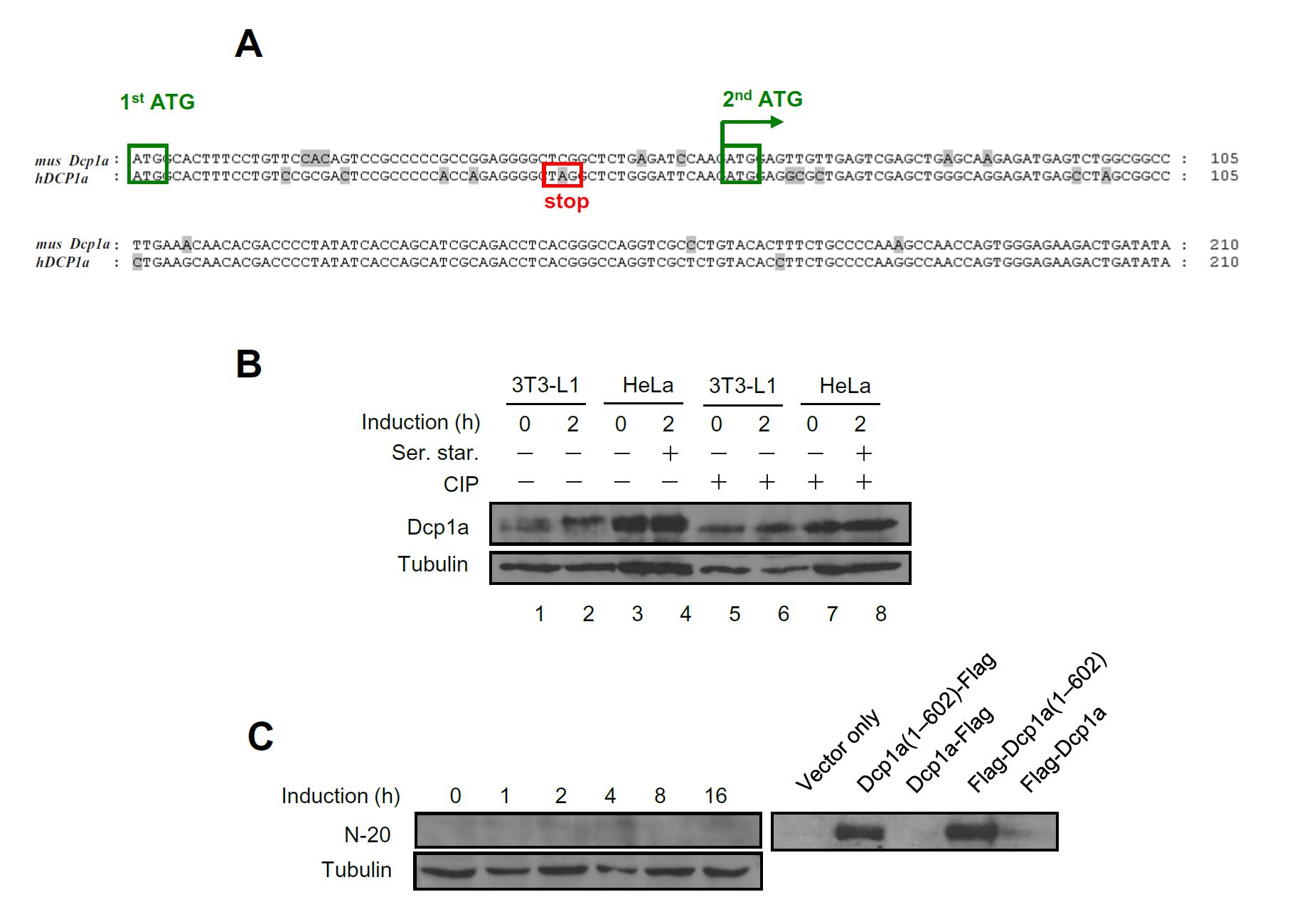

Supplement: Figure S1 — Identification of a possible translation initiation codon in mouse DCP1a. (A) The nucleotide sequences of Dcp1a in mouse and human both contain two start codons that are 60 nucleotides apart. Human DCP1a has an in-frame stop-codon for the first AUG, but mouse Dcp1a does not. (B) Comparison of endogenous Dcp1a expression in mouse and human cells. 3T3-L1 (mouse) cell cultures were induced to differentiate for 0 and 2 h (lanes 1 and 2). HeLa (human) cells were serum starved overnight followed by induced for 2 h with Gibco Qualified FBS (lane 4), and untreated cells served as a control (lane 3). The remaining lanes (lanes 5–8), show cell extracts from lanes 1–4 were treated with 10 U CIP at 37°C for 1 h. (C) A rabbit polyclonal antiserum (N-20) specifically recognizing the first 20 amino acids of the predicted full-length mouse Dcp1a was generated. Antibody specificity (right panel) was shown by transient transfection of the vector control, Flag-tagged full-length Dcp1a (1–602), or Flag-tagged Dcp1a that begins at the second AUG (Dcp1a). Expression of endogenous Dcp1a (left figure) in 3T3-L1 cells was not detected by the antiserum. This implies that the translation of the Dcp1a mRNA started from the second AUG in the sequence. (TIF) [file pone.0061697.s001.tif]

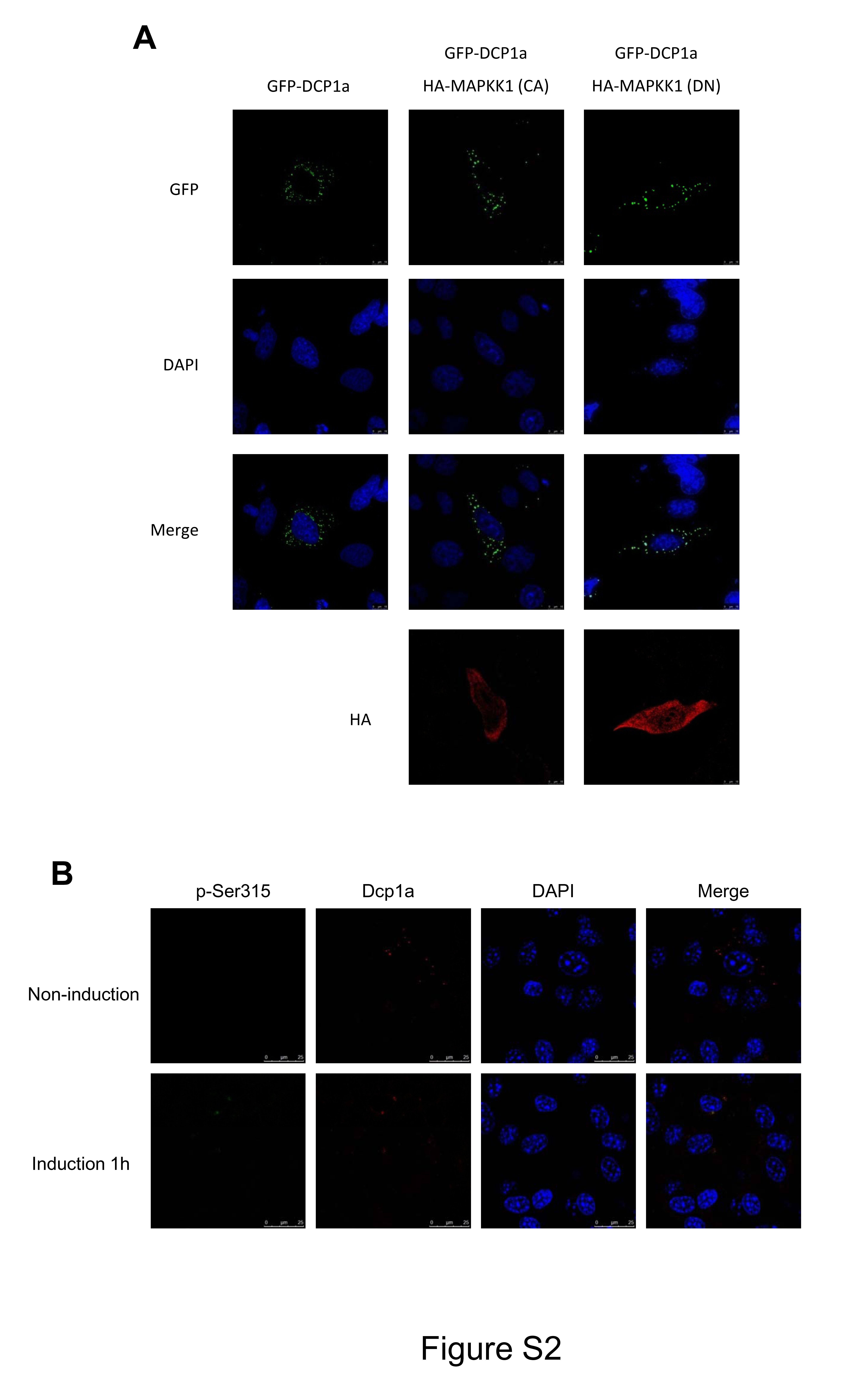

Supplement: Figure S2 — Indirect immunofluorescence of Dcp1a in cells. (A) HeLa cells cultured in DMEM with 10% FBS on 3.5-cm dishes were transfected with GFP-Dcp1a (co-transfection with CA or DN MAPKK1). Cells were immunostained 1 day after transfection. Cells were rinsed briefly in PBS and fixed with 2% formaldehyde in PBS at room temperature for 20 min. HA-tagged MAPKK1 was stained with anti-HA followed by Alexa 594–conjugated anti-mouse, and the nuclei were stained with DAPI. The cells were visualized with confocal laser microscopy. (B) Two days after reaching confluency, cultures of 3T3-L1 preadipocytes were non-induced or induced with an induction cocktail FMDI for 1 h. Cells were fixed and immunostained with anti-Dcp1a followed by Alexa 594-conjugated anti-mouse and anti-p-Ser315 followed by Alexa 488-conjugated anti-rabbit, and the nuclei were stained with DAPI. The cells were visualized with confocal laser microscopy. (TIF) [file pone.0061697.s002.tif]

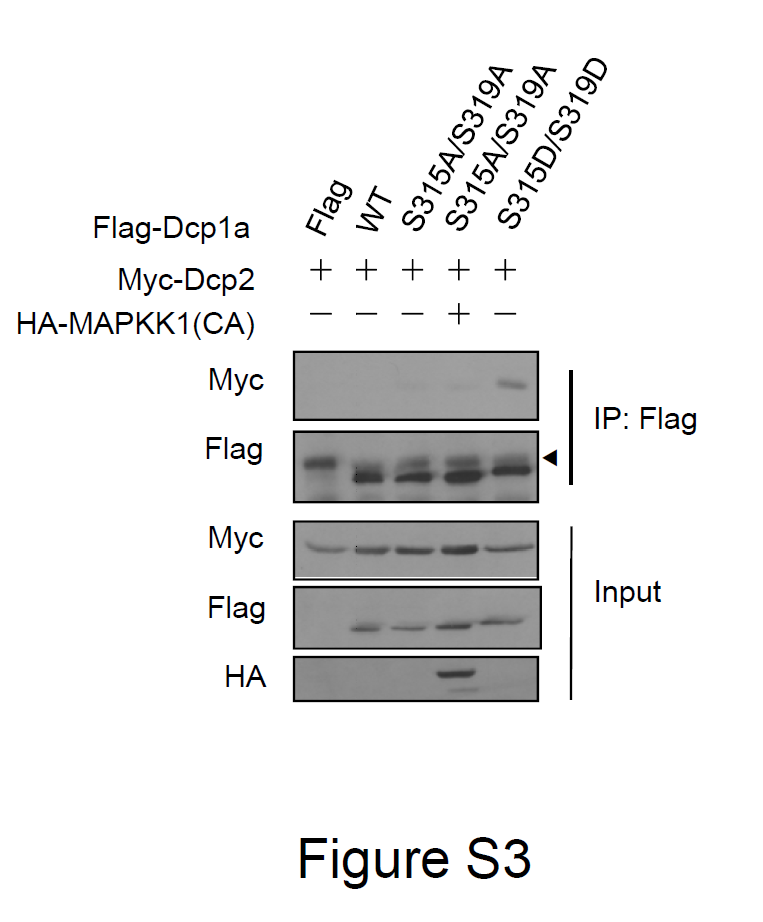

Supplement: Figure S3 — ERK signal can not further enhance the interaction between S315A/S319A Dcp1a mutant and Dcp2. HEK 293T cells were transfected with Flag-Dcp1a(wild type [WT]), HA-tagged CA MAPKK1, Flag-Dcp1a(S315A/S319A), Flag-Dcp1a(S315D/S319D), and Myc-Dcp2 as indicated. Protein complexes immunoprecipitated by anti-Flag wereanalysed by western blotting with indicated antibodies. The arrowhead indicates the non-specific band. (TIF) [file pone.0061697.s003.tif]

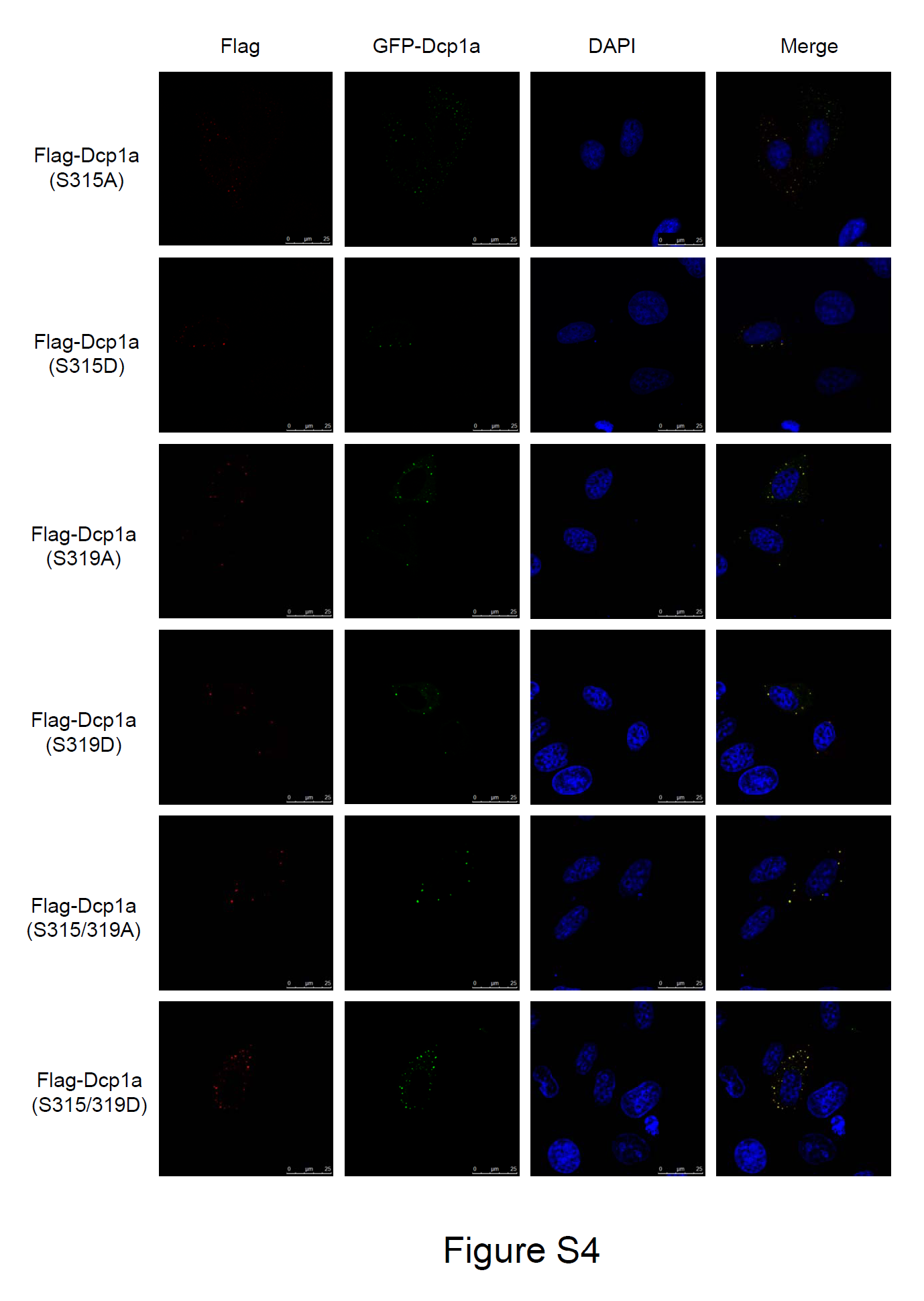

Supplement: Figure S4 — The similar cellular distribution of WT, S315A/S319A, and S315D/319D Dcp1a. HeLa cells were transfected with GFP-Dcp1a combined with Flag-Dcp1a mutant as indicated. Cells were immunostained 1 day after transfection with anti-Flag followed by Alexa 594–conjugated anti-mouse, and the nuclei were stained with DAPI. The cells were visualized with confocal laser microscopy. (TIF) [file pone.0061697.s004.tif]

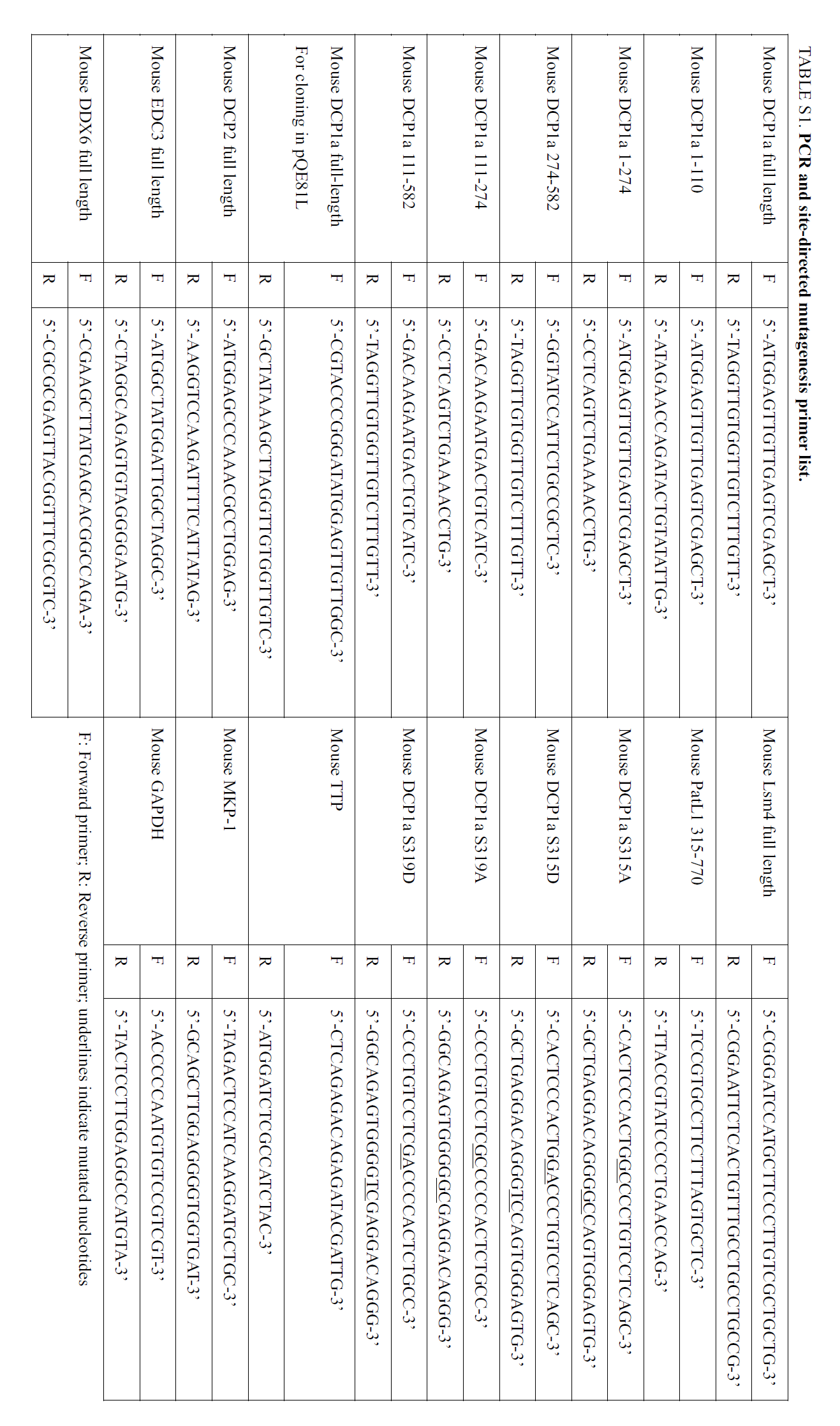

Supplement: Table S1 — PCR and site-directed mutagenesis primer list. (TIF) [file pone.0061697.s005.tif]
